# Prognostic Value of HALP Score in Rheumatic Mitral Stenosis: A Long‐Term Follow‐Up Study

**DOI:** 10.1002/clc.70324

**Published:** 2026-04-20

**Authors:** Sundas Adnan Butt

**Affiliations:** ^1^ Fatima Jinnah Medical University Lahore Pakistan

To Editor

The article, “Prognostic Value of HALP Score in Rheumatic Mitral Stenosis: A Long‐Term Follow‐up Study” [1] offers a good perspective on how the hemoglobin, albumin, lymphocyte, and platelet (HALP) score can be one of the prognostic biomarkers. Due to the growing interest in inflammation‐based indexes in cardiovascular disease, it is worth praising the authors to study this convenient and economical index. Nevertheless, some limitations should be identified.

First, there is no comparison of HALP with other well‐known inflammation‐based or nutritional indices like the Prognostic Nutritional Index (PNI), neutrophil‐to‐lymphocyte ratio (NLR), or platelet‐to‐lymphocyte ratio (PLR). Devoid of such comparisons, it is not clear whether HALP provides incremental prognostic information or is a simple mirror of comparable underlying inflammatory actions that are represented by existing markers. Research shows that both HALP and PNI play overlapping roles as predictors [2], which makes the comparison of the two results imperative. Likewise, another article [3] emphasized the need to measure new indices in addition to the existing risk markers to ascertain their complementary clinical value. The absence of such analysis in the current study impairs the positioning of HALP in the existing risk stratification models.

Second, acute clinical conditions like infections or stress reactions may interfere with the elements of HALP. As lymphocyte count, platelet count, and albumin levels are very sensitive to acute inflammatory conditions, the baseline HALP levels can be inaccurate when evaluating the severity of chronic diseases in patients with rheumatic mitral stenosis. This leads to the risk of misclassification bias. The association between HALP score and infection in acute ischemic stroke patients [4] shows evidence that infection has a significant effect on HALP, and thus, it impacts the reliability of the prognostics used. Subsequent studies need to involve screening for acute infection, the use of inflammatory biomarkers like C‐reactive protein or procalcitonin, and repeated measurements to be able to stabilize baseline values of HALP.

Third, the type of clinical outcomes is not adequately separated in the study, as it results mainly by all‐cause mortality or composite endpoints. This limits the specificity of the results, since it has not been established whether HALP is a predictor of cardiovascular mortality in particular or merely a sign of systemic illness and frailty. Previous studies indicate that prognostic performance of HALP might differ with either the definition of outcomes as mortality, complication, or disease‐specific events [5]. On the same note, cardiovascular versus non‐cardiovascular endpoints have been found to be significant in valvular surgery population studies. In the absence of this stratification, clinical interpretation of HALP in rheumatic mitral stenosis is not clear. Future research must examine cause‐specific outcomes such as cardiovascular mortality, heart failure‐related hospitalization, thromboembolic events, and should consider using competing risk models to enhance accuracy.

Although the research adds to the accumulation of literature on the prognostic markers based on inflammation, the following limitations indicate the necessity to use more in‐depth analytical methods. It will be important to address the concerns with the help of comparative biomarker analysis, scrupulous management of acute inflammatory conditions, and more specific outcomes measurement to determine the actual clinical value of the HALP score in rheumatic mitral stenosis.

## Funding

The author has nothing to report.

## Conflicts of Interest

The author declares no conflicts of interest.

## 1 Declaration of Generative AI and AI‐Assisted Technologies in the Writing Process

After the preparation of this Letter, the author used OpenAI's ChatGPT in order to enhance the readability and language of the manuscript. After using this tool, the author reviewed and edited the content as needed and takes full responsibility for the content of the publication.

## Data Availability

Data sharing is not applicable to this article as no data sets were generated or analyzed during the current study.

## References

[1] A. F. Kaya , G. Ayhan , V. Can , E. Özbek , L. Dursun , and Ö. Kümet , “Prognostic Value of HALP Score in Rheumatic Mitral Stenosis: A Long‐Term Follow‐Up Study,” Clinical Cardiology 49, no. 4 (2026): e70287, 10.1002/clc.70287. 41891398 PMC13045295

[2] I. Koyuncu and E. Koyun , “Relationship Between HALP and PNI Score With 1‐Month Mortality After CABG,” Frontiers in Nutrition 11 (2024): 1489301, 10.3389/fnut.2024.1489301. 39555199 PMC11563828

[3] S. Söner , T. Güzel , A. Aktan , et al., “Prognostic Value of Hemoglobin, Albumin, Lymphocyte, Platelet (HALP) Scores in Patients With Non‐Valvular Atrial Fibrillation: Insights From the AFTER‐2 Study,” BMC Cardiovascular Disorders 25, no. 1 (2025): 528, 10.1186/s12872-025-04993-1. 40684097 PMC12275269

[4] Y. Demir , B. Yamak , S. Sevinç , et al., “HALP Score as a Prognostic Biomarker in Tricuspid Valve Surgery: Association With In‐Hospital and Long‐Term Mortality,” Journal of Inflammation Research 18 (2025): 10637–10649, 10.2147/JIR.S517577. 40799800 PMC12341553

[5] U. Ozturk , S. Nergiz , and O. Ozturk , “The Association Between HALP Score and Infection in Acute Ischemic Stroke Patients,” Journal of Stroke and Cerebrovascular Diseases 33, no. 11 (2024): 107929, 10.1016/j.jstrokecerebrovasdis.2024.107929. 39159902

